# A Monte Carlo-based framework enhances the discovery and interpretation of regulatory sequence motifs

**DOI:** 10.1186/1471-2105-13-317

**Published:** 2012-11-27

**Authors:** Phillip Seitzer, Elizabeth G Wilbanks, David J Larsen, Marc T Facciotti

**Affiliations:** 1 Department of Biomedical Engineering, One Shields Ave, University of California, Davis, CA 95616, USA; 2 Genome Center, One Shields Ave, University of California, Davis, CA 95616, USA; 3 Microbiology Graduate Group, One Shields Ave, University of California, Davis, CA, 95616, USA

**Keywords:** Motif, Monte Carlo, ChIP-seq, ChIP-chip, Comparative genomics, MEME, STAMP, TFB

## Abstract

**Background:**

Discovery of functionally significant short, statistically overrepresented subsequence patterns (motifs) in a set of sequences is a challenging problem in bioinformatics. Oftentimes, not all sequences in the set contain a motif. These non-motif-containing sequences complicate the algorithmic discovery of motifs. Filtering the non-motif-containing sequences from the larger set of sequences while simultaneously determining the identity of the motif is, therefore, desirable and a non-trivial problem in motif discovery research.

**Results:**

We describe MotifCatcher, a framework that extends the sensitivity of existing motif-finding tools by employing random sampling to effectively remove non-motif-containing sequences from the motif search. We developed two implementations of our algorithm; each built around a commonly used motif-finding tool, and applied our algorithm to three diverse chromatin immunoprecipitation (ChIP) data sets. In each case, the motif finder with the MotifCatcher extension demonstrated improved sensitivity over the motif finder alone. Our approach organizes candidate functionally significant discovered motifs into a tree, which allowed us to make additional insights. In all cases, we were able to support our findings with experimental work from the literature.

**Conclusions:**

Our framework demonstrates that additional processing at the sequence entry level can significantly improve the performance of existing motif-finding tools. For each biological data set tested, we were able to propose novel biological hypotheses supported by experimental work from the literature. Specifically, in *Escherichia coli*, we suggested binding site motifs for 6 non-traditional LexA protein binding sites; in *Saccharomyces cerevisiae,* we hypothesize 2 disparate mechanisms for novel binding sites of the Cse4p protein; and in *Halobacterium* sp. NRC-1, we discoverd subtle differences in a general transcription factor (GTF) binding site motif across several data sets. We suggest that small differences in our discovered motif could confer specificity for one or more homologous GTF proteins. We offer a free implementation of the MotifCatcher software package at http://www.bme.ucdavis.edu/facciotti/resources_data/software/.

## Background

The problem of discovering functional DNA or protein subsequences (motifs) in biological sequences data has driven the development of numerous motif-finding tools. Two approaches dominate motif-finding algorithms: enumerative word-based methods and probabilistic sequence models that optimize model parameters by applying expectation-maximization techniques or Bayesian inference [1]. These ideas were first applied in a handful of tools developed more than twenty years ago [2-6]. Most subsequently developed motif-finders recapitulate these approaches with subtle variations, and report incremental improvements in motif detection. Motif searches have become more sophisticated in terms of the patterns they can recover - where we could once only discover identical sequence matches, we may now discover gapped, palindromic, and degenerate subsequences [7-11]. The incorporation of another type of meaningful prior information to the motif search may also improve motif detection: motif searches have improved to incorporate background sequence models, phylogenetic information, experimental data, and other large-scale prior information [12-18].

**Figure 1:**
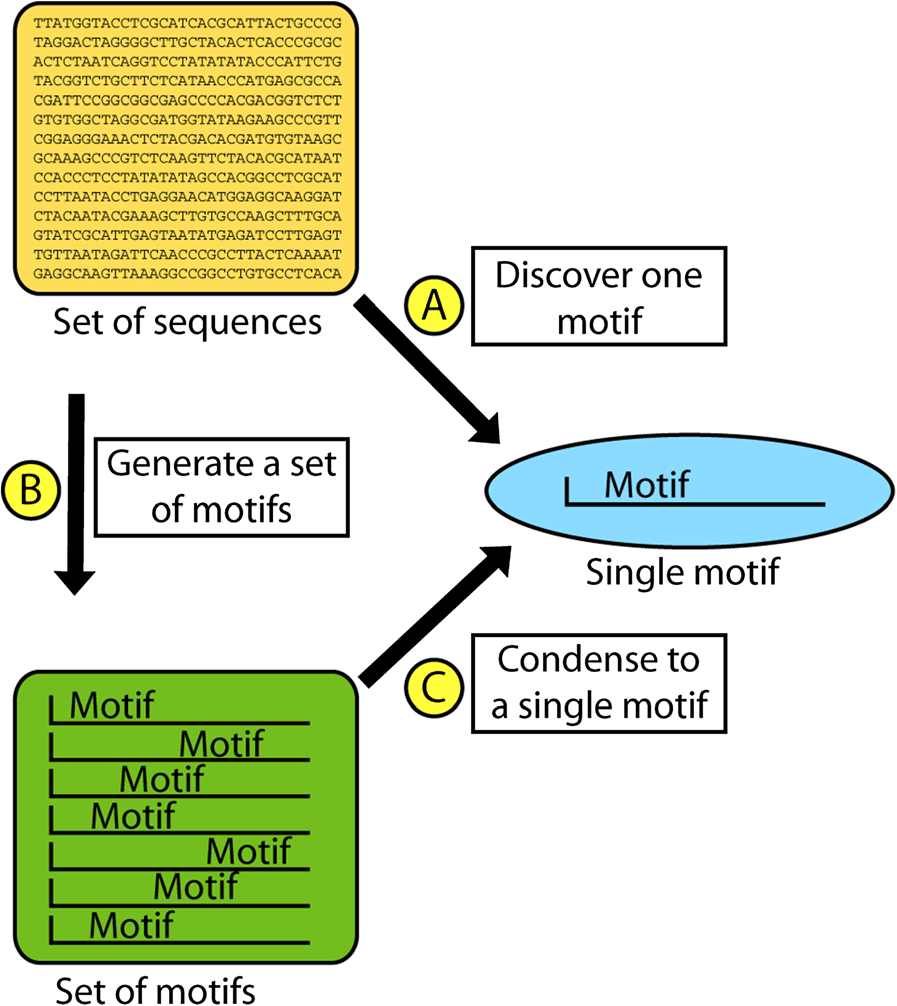
**Major Focuses in Motif-finding research.** The problems of discovering a significant subsequence motif from a set of sequences (**A**), discovering a candidate list of redundant subsequence motifs from a set of sequences (**B**), and discovering a non-redundant, condensed motif from a set of redundant subsequence motifs (**C**) are interrelated. Integrated motif-discovery frameworks seek to perform both (**B**) and (**C**), often by combining several tools designed to retrieve a single motif (**A**). In this description, discovering “one motif” or a “set of motifs” refer to descriptions of a single functionally meaningful phenomenon - in principle, multiple independent functionally meaningful motifs may exist in a sequence data set, however we avoid representing this case in the above figure to avoid confusion.

Interest in the problem of motif discovery has inspired work on two related problems: (1) the generation of a set of candidate motifs from a data set of sequences, and (2) the problem of meaningfully condensing a set of motifs into a single non-redundant motif (Figure 1). To generate a set of candidate motifs from a data set of sequences, researchers have entered the same input set of sequences into several different motif-finding tools [19-21], as well as applied one or more motif-finding tools to different random subsets of the input set of sequences [19]. To meaningfully condense a set of motifs to a single motif, various statistical tests [19], linkage tree construction and parsing [21], and other motif compression approaches (such as that of the “familial binding profile” [22-24] and “metamotif” [25]) have been developed. The relatedness of the problems of motif discovery, candidate motif set creation, and motif set condensation to a single motif has inspired the creation of integrated frameworks [21,26]– tools that first generate a candidate set of motifs (Figure 1B), and then meaningfully parse this set of motifs to produce a meaningful representative motif (Figure 1C).

Here we present MotifCatcher: an integrated framework for motif discovery. Our framework utilizes random sampling of the input data set to generate a candidate list of motifs. We extend upon previous random sampling approaches by developing and applying 3 different random sampling-based candidate motif-generating protocols. We examine the effects of each approach in turn, and discover the best results with a novel position-specific iterative process. We create a linkage tree of these candidate motifs by integrating with existing motif tree generating software, which allow for 6 possible motif comparison and evaluation metrics. The user may decide to segment the linkage tree at various thresholds in order to condense individual motif leaves into a single, aggregate motif. Generation and parsing of a linkage tree of motifs has been used previously [21], however, in that case, the condensation of a set of motifs into a single motif focused on similarities and differences among the individual candidate motifs. An average motif is computed based on an analysis of the output motifs, without consideration of the subsequences from which they are created. The transfer of aligned subsequences to motif representation incurs a loss of information: a motif describes only the frequency of individual sequence elements at particular positions in a set of aligned subsequences, discarding the actual subsequences from which these sequence elements arise. Our motif condensation approach is based on the frequency of occurrence of individual subsequences in the output motifs, which allows us to retain dependent positional relationships between sequence elements in each subsequence string, and explicitly filter out subsequences erroneously included in the set of output motifs.

For motif-finding to operate smoothly, sequence data sets should be assembled with as high a ratio of motif signal sequence to background sequence as possible [27]. We accomplish this task by randomly sampling the whole data set, and searching for motifs in these random subsets, with the expectation that certain subsets will have a high ratio of motif signal to background sequence. Sequence entries are incorporated in sequence data sets because they are suspected to contain a significant motif, but this need not always be the case - For example, ChIP-chip (Chromatin Immunoprecipitation with hybridization by microarray) and ChIP-seq (Chromatin Immunoprecipitation with massively parallel sequencing) experiments generate sequence data sets reflecting DNA binding events of a particular protein of interest, but not all reported binding events necessarily involve the presence of a unique motif (this may arise as a consequence of indirect protein-protein interactions accompanying protein-DNA associations [28]). Sequence data sets with non-motif-containing sequence entries also arise from non-experimental analyses, such as the comparison of upstream regions of one or more putatively co-regulated genes across a set of related organisms [29]. MotifCatcher’s emphasis on random sampling allows it to find significant motifs in data sets containing a very large number of non-sequence-containing entries (Additional file 1: Figure S1 and Additional file 2). In this regard, MotifCatcher offers an advantage over integrated motif discovery frameworks that only aggregate the output of many different motif-finding tools [20,21], because these motif-finding tools may fail to find motifs in data sets containing many non-motif-containing sequence entries. It is worth mentioning that a number of tools have been developed to discover motifs exclusively in ChIP data [30,31]. MotifCatcher is appropriately applied to ChIP data, however the generality of its algorithm makes it applicable to any sequence data set, and especially effective in discovering motifs in sets of sequences where a large number of sequence entries do not contain a motif.

To evaluate the performance of MotifCatcher, we explored several published and unpublished ChIP-chip and ChIP-seq data sets. Our investigations incorporated organisms from all three domains of life and highlighted biological phenomena with associated motifs spanning a large range in length, degeneracy, and prevalence among input sequence entries. We developed two implementations of the MotifCatcher approach, each employing a different mainstream motif-finding program (expectation maximization via the MEME Suite [5,27], and Gibbs sampling via a Gibbs recursive sampler [4,32]). In every investigation, we compared the performance of the MotifCatcher extension of the motif finder to the performance of the motif finder alone. Additionally, we evaluate MotifCatcher’s output motif tree to yield meaningful biological insights.

## Results

### Theory and Motivation

If an input set of sequence entries is corrupted with non-motif containing sequence entries, from the standpoint of motif discovery, these non-motif containing sequences do not belong in the dataset – ideally, we would like to remove these sequences from the data set prior to carrying out a motif search. However, without knowing the identity of the motif or the sequence entries that contain them, we cannot remove these sequences directly. This problem has been addressed previously, in the MEME ZOOPS (zero or one occurrence per site) algorithm. In the MEME algorithm, sequence data is thought of as the product of a finite mixture model with unknown parameters [5]. Using an expectation maximization algorithm, the values of the parameters are estimated based on the observed sequences data. Using the ZOOPS generalization, sequence entries are each assigned a prior probability that they contain a motif. The posterior probabilities are determined during the expectation maximization process [7]. Low posterior probabilities therefore may effectively remove sequence entries from the data set. This protocol is very effective when the true motif stands out from the background, however it is more challenging to retrieve the motif in cases where the motif is obscured by a large amount of noisy background sequence. Instead of estimating parameters based on a collective input body of sequences, we explore the more extreme possibility of excluding these sequences entirely – effectively, assigning to a large set of sequences a prior probability of zero, even before any parameter estimation of the data set has occurred.

In order to discover which sequences contain motifs and which do not, we use a random sampling approach combined with motif searches to determine a set $\bar{R}$ of candidate “related subsets” R. Certain R will not yield significant motifs at all, and are discarded. Among the R that yield a significant motif, certain highly similar motifs will tend to be re-discovered from many different random seeds. Candidate significant motifs are hierarchically clustered and joined by linkage tree, where frequently re-occurring motifs from $\bar{R}$ naturally cluster into highly dense branches. In our analyses, we discovered that convergence to a common point (similar motif) from many different starting states (random subsets of the data set) is often an indication that the motif is meaningful. Our emphasis on convergence to a common end point from many random starting states bears much similarity to a classic Monte Carlo Markov Chain algorithm [33].

### Algorithm

**Figure 2:**
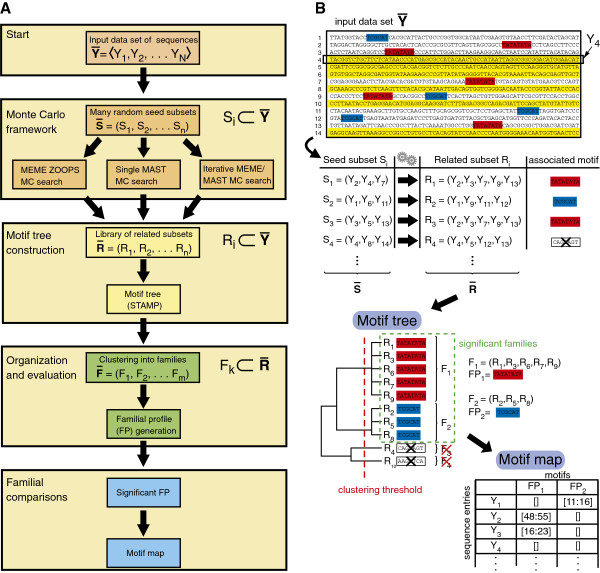
**Flowchart of the MotifCatcher algorithm.** (**A**) The MotifCatcher pipeline may be conceptually divided into 4 processing stages, starting with an input data set of sequences $\bar{Y}$: (1) A set of random subsets of $\bar{Y},\;\bar{S}=\left(S_{1},S_{2},\ldots S_{n}\right)$, where each $S_{i}\subset \bar{Y}$, are converted into a set of related subsets $\bar{R}=\left(R_{1},R_{2},\ldots R_{n}\right)$, using one of the three related subset determination protocols. (2) $R_{i}\subset \bar{R}$ (with statistically significant associated motifs) are organized into a branching diagram according to the similarity of their motifs using the STAMP platform. (3) R_i_ with highly similar associated motifs are clustered together into families F_i_ according to a user-determined clustering threshold. (4) A representative motif of each family, the familiar profile (FP), is computed, as well as a motif map of the subsequences from all sequence entries from $\bar{Y}$ used to construct different FPs. (**B**) Application of the MotifCatcher algorithm to a toy data set of 14 sequence entries, all 68 nt in length. Two significant motifs (TATATATA, highlighted in red, and CTGCAT, highlighted in blue) are recovered from a MotifCatcher search of 10 seeds. In this example, 6 of the 14 sequence entries do not contain a significant motif (highlighted in yellow). 8 of the 10 seeds converge to a meaningful motif (three examples illustrated in random subsets S_1_, S_2_, and S_3_), and 2 of the 10 seeds do not (exemplified in random subset S_4_). Seed subsets containing motif-rich sequence entries converge to related subsets with meaningful motifs, seed subsets lacking motif-rich sequence entries do not converge to related subsets with meaningful motifs (and so are discarded). Conversion of a seed subset S_i_ to a related subset R_i_ is achieved by one of three different protocols, here represented by gears, and arrows pointing from each enumerated seed subset to its resultant related subset. The 10 related subsets are organized into a motif tree, thereupon the 2 related subsets lacking meaningful motifs are discarded, and familial profiles are determined for the 2 meaningful families. A motif map structure maps familial profiles back to the input data set $\bar{Y}$.

The algorithmic approach of MotifCatcher is summarized in Figure 2. The following describes an implementation specifically tailored for use with the MEME Suite, though in principal any motif-discovery algorithm could replace MEME, and any motif-scanning algorithm could replace MAST (Motif Annotation Search Tool) [34]. The MEME Suite was chosen based on its confirmed effectiveness, usability, and prevalence of use in the bioinformatics community. In addition to the described MEME/MAST workflow, we developed an analogous workflow with the Gibbs recursive sampler replacing MEME. For a longer alternative version of this algorithm, please see Additional file 1: S2 and Additional file 2. Screenshots of our implemented GUI framework are available in Additional file 1: Figure S2 and Additional file 2.

#### Monte Carlo framework

From an input data set of N sequence entries $\bar{Y}=\left(Y_{1},Y_{2},\ldots Y_{N}\right)$, n random seed subsets $\bar{S}=\left(S_{1},S_{2},\ldots S_{n}\right)$ are extracted, where each $S_{i}\subset \bar{Y}$. ‘n’ is a user-specified value, and should be selected according to the size of $\bar{Y}$ and the expected number of $Y_{i}\subset \bar{Y}$ that is thought to contain a subsequence instance of a significant motif. Three alternative schemes are available to create a library of related subsets $\bar{R}$ from the set of seed subsets $\bar{S}$, applied to each $S_{i}\subset \bar{S}$: (1) MEME ZOOPS MC (MotifCatcher) search: A MEME ZOOPS search is applied to S_i_, and sequence entries that contain subsequences included in the construction of the MEME ZOOPS-produced motif comprise R_i_. (2) Single MAST MC (MotifCatcher) search: A MEME ZOOPS search is applied to S_i_, and the MEME ZOOPS-produced motif is scanned over $\bar{Y}$ using the MAST. All sequence entries in $\bar{Y}$ that contain a significant subsequence match to the preliminary motif comprise R_i_. (3) Iterative MEME/MAST MC (MotifCatcher) search: A MEME ZOOPS search is applied to S_i_ producing a motif M. Using MAST, $\bar{Y}$ is scanned for the motif M. All sequence entries in $\bar{Y}$ that contain a significant match are collected. These sequences constitute the modified seed S_i_'. MEME ZOOPS is then applied to the modified seed S_i_', to produce a modified motif, M’ which is scanned over $\bar{Y}$ with MAST as before. This iterative search continues until convergence: a MEME search of a modified seed S_i_^mod^ produces a motif M_mod_, and a MAST search of M_mod_ over $\bar{Y}$ finds subsequence instances of M_mod_ in (and only in) S_i_^mod^. The sequence entries in S_i_^mod^ comprise R_i_. Typically, the iterative search (scheme 3) is to be preferred, as it will tend to converge upon meaningful motifs more often than the non-iterative and single-scan approaches (schemes 1 and 2).

#### Motif tree construction

A branching diagram is constructed comparing the relative similarity of motifs associated with each of the different related subsets R_i_ in $\bar{R}$ (motif tree). Some of the R_i_-associated motifs may not be statistically significant. Thus, all $R_{i}\subset \bar{R}$ with an associated motif with a high E-value (typically, this value should be no larger than 0.01) are excluded from further analysis. As $\bar{R}$ may be very large, all R_i_ except a small subset of $\bar{R}$ with the lowest R_i_-associated motif E-values may be excluded. The STAMP platform (Similarity, Tree-building, and Alignment of DNA Motifs and Profiles) [23,24] is utilized to organize the remaining $R_{i}\subset \bar{R}$ into a distance tree according to similarities of their R_i_-associated motifs. The pairwise distance between two motifs is computed in a column-by-column fashion using one of several available statistical metrics that can be selected by the user [23]. After a pairwise distance has been computed between all R_i_-associated motifs, the distance tree is assembled using either an unweighted pair group method with arithmetic mean (UPGMA) or self-organizing tree algorithm (SOTA), according to the user’s preference.

#### Organization and evaluation

The $R_{i}\subset \bar{R}$ represented in the motif tree will naturally cluster into groups according to the similarity of their R_i_-associated motifs. Each motif family F_i_ is a collection of R_i_ (grouped by similarity among their R_i_-associated motifs). The motif tree is therefore defined by a set of m non-intersecting motif families $\bar{F}=\left(F_{i},F_{2},\ldots F_{m}\right)$. The division of the motif tree into a set of non-intersecting motif families requires that a clustering threshold be imposed upon the $R_{i}\subset \bar{R}$ represented in the tree. Each motif family F_k_ is a collection of R_i_, and each R_i_ is a collection of sequence entries taken from the whole input set $\bar{Y}$. The set of motif families $\bar{F}=F_{1},F_{2},\ldots F_{m}$ is determined based entirely on the similarity of R_i_-associated motifs, without regard to the sequence entries from which subsequences are drawn to create these R_i_-associated motifs. The motif family F_k_ can be described by a singular characteristic motif, which we refer to as a “familial profile” (FP). Among the collection of R_i_ that forms F_k_, some sequence entries will be re-discovered more frequently than others. An FP is generated for each F_k_ according to a user-selected FP frequency threshold. To compute the FP, sequence entries Y_j_ that re-occur among the related subsets in a motif family with frequency of greater than or equal to the FP frequency threshold are collected and a motif is generated from these sequence entries using a MEME OOPS (one occurrence per site) model. To compare the occurrence of different motif families over the set of sequence entries, a “MotifMap” matrix is created. This matrix compares the input sequence entries (rows) to significant FPs (columns). When an instance of FP, the familial profile derived from family F, is found in a particular input sequence entry Y, the coordinates that this instance spans within the sequence entry are noted at position (row, column) in the matrix.

### Tests on biological data

#### LexA binding in *E. coli*

The absence of clearly identifiable motifs in large subsets of ChIP-chip and ChIP-seq data is a common occurrence. This can result from experimental error or protein binding to degenerate or motif-free sites [28]. To test MotifCatcher’s ability to discover a motif in a data set with a large number of motif-free sites, we reanalyzed ChIP-Chip data collected by Wade *et al.*, [35] who identified 49 binding sites for the well-studied regulatory protein LexA in *E. coli* MG1655. Twenty-five of the binding sites were consistent with previous experimentally determined targets (termed type I sites) and 24 were novel sites. Wade *et al*. scanned the novel binding sites for a significant match to the canonical TACTG(TA)_10_CAGTA LexA motif [36]. Five of these sites were found to have a canonical LexA motif (and thus termed type II sites), and 19 were determined not to have a canonical LexA motif (termed type III sites) [37]. We sought to test whether or not MotifCatcher could improve upon the previous interpretation of this dataset. In our reanalysis of the ChIP-chip data, we assembled a sequence data set based on the 49 LexA binding sites reported by *Wade et al.*, and an additional sequence data set with random genomic sites substituted for the type III sites. A motif search was carried out on both data sets with MEME ZOOPS, recursive Gibbs sampling, MEME ZOOPS MC, single MAST MC, and an iterative MEME/MAST MC.

**Figure 3:**
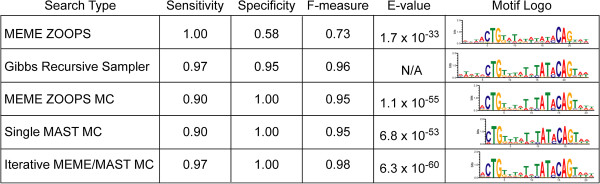
**Comparison of motif finders in their ability to identify true LexA binding sites.** A dataset of constructed of 30 LexA binding sites and 19 random 1000-mers taken from around the *E. coli* genome was evaluated with 2 popular motif finders (MEME ZOOPS and Gibbs recursive sampler) and each of the 3 available MotifCatcher options (MEME ZOOPS MC, Single MAST MC, Iterative MEME/MAST MC). MotifCatcher results were selected with an FP Threshold value based on the lowest E-value reported. Results from all 3 MotifCatcher frameworks had better specificity (column 3) than the MEME ZOOPS and Gibbs recursive sampler results, and the iterative search (Iterative MEME/MAST MC) had the best F-measure of all 5 options. The motifs produced by the Gibbs recursive sampler results and all 3 MotifCatcher runs demonstrated a pronounced T-TATA upstream of the CAG (column 5) not seen in the MEME ZOOPS results. This difference represents a de-emphasis in palindromicity of the motif, suggesting a directionality of LexA protein binding *in vivo*. Note that the MotifCatcher runs produced motifs with E-values 20–27 orders of magnitude than the MEME ZOOPS result, which corresponded to an improvement in F-measure of about 0.25.

For the data set with random sites substituted for type III sites, type I and II sites are true positives (genomic locations where the LexA protein binds), and random sites are true negatives (genomic locations where the LexA protein does not bind). The erroneous discovery of a LexA binding site at a random genomic location is therefore a false positive, and the failure to discover a LexA binding site at a genomic location where the LexA protein binds is a false negative. The most accurate binding site motif representation should be built from only true binding sites (perfect specificity), and include all true binding sites (perfect sensitivity). The MEME ZOOPS and recursive Gibbs sampler approaches produce only one final output motif, while the three MC searches offer alternative versions of output motifs, according to the FP threshold value. Selecting a higher FP threshold value produces a conservative estimate of the motif, while a lower value may include more degenerate subsequences, at the cost of erroneously incorporating more non-motif-containing sequences. In our analysis, we explored the whole range of FP threshold values (from 0 to 1), and discovered that the statistically best F-measure always correlated with the best E-value (the F-measure is defined as the harmonic mean of sensitivity and specificity). (Additional file 1: Figure S3, S5 and Additional file 2). This finding corroborates previous work that has suggested that E-value may be a better statistical measure to optimize than the more traditional measure of information content, which is the objective of the MEME ZOOPS protocol [5,7,38]. A comparison of the various motif-finding strategies applied to this data set revealed that each of the three MC searches demonstrated comparable performance to the Gibbs recursive sampler and outperformed MEME significantly. A significant difference in the actual motif profiles determined by the MotifCatcher and Gibbs sampling approaches further suggests a distinct directionality of the motif not reported by the MEME ZOOPS search (Figure 3).

**Figure 4:**
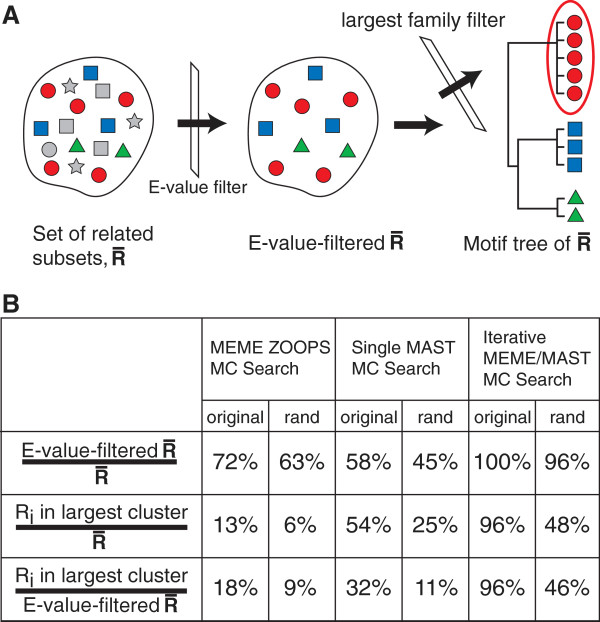
**Motif tree-building protocol and application to ChIP-chip derived LexA binding sites in E Coli.** (**A**) Illustration of MotifCatcher’s motif tree building protocol. In a set of related subsets $\bar{R}$, all Related Subsets R with associated motifs with an E-value greater than E-value threshold are filtered out (when using the MEME platform or any other motif finder that uses E-value as a measure of statistical significance), and the remaining $R_{i}\subset \bar{R}$ are organized into a motif tree. The largest motif family describes the motif most likely to be biologically significant. Depending on the input sequence data set, smaller clusters may also represent biologically significant motifs. In this example, R_i_-associated motifs are represented as simple polygons (circles, squares, triangles, stars). Gray polygons represent R_i_-associated motifs with an E-value above the E-value threshold, and so are eliminated in the initial filtration step. The remaining colored polygons are organized into a motif tree, in which the red circles form the largest cluster (circled in red on tree). In this toy system, there are 17 total $R_{i}\subset \bar{R}$, of which 10 pass the E-value filter, of which 5 segregate into the largest cluster. Comparative ratios from the LexA study shown in the table in (**B**) reveal that regardless of the related subset determination protocol, it was always easier to recover the LexA motif from the original data set versus the set with non-traditional LexA binding sites replaced by random sites. In all trials, the largest cluster motif recapitulated the canonical LexA motif.

The MEME ZOOPS searches produced motifs similar to the canonical LexA-binding site motif (with similar E-values) for both data sets. Subsequences from both type III sites and random sites were included in the creation of each respective binding site motif at approximately the same rate (original data set: 40 of the 49 subsequences included, 10 of which were type III sites; random sites substituted data set: 38 of the 49 subsequences included, 8 of which were random sites). From MotifCatcher’s tree-building process, additional meaningful quantities emerge in both the total number of significant related subsets, and the fraction of R_i_ that comprise the largest motif family (Figure 4A). A comparison of these values for the original data set versus the random sites substituted data set reveals that the original data set exhibits both (1) a significantly greater proportion of total R_i_ runs with an associated motif with a significant E-value, and (2) of these significant R_i_, a greater fraction of these have an associated motif similar to the canonical LexA-binding site motif (Figure 4B). For all three types of MC searches, it was easier to discover the canonical LexA motif when type I and type II sites were supplemented with type III sites instead of random sites.

An analysis of the degenerate motif subsequence matches suggested by MotifCatcher revealed that MotifCatcher consistently preferred a single subsequence from each sequence entry to incorporate in the LexA motif from the type III sites (data not shown). Conversely, MotifCatcher typically did not settle on a single subsequence match from the random sites data set. As each sequence entry was derived from a single ChIP-chip derived LexA binding site, only one subsequence from the set ought to represent the genuine LexA binding site location. In this way, the type III sites were again distinguished from randomness (data not shown). An investigation of the degenerate subsequence matches suggested by MotifCatcher revealed that 6 of the 19 type III sequence entries contained subsequences that were identical to the canonical site (Table 1), but with one or more CTG half-site motifs replaced by ATGs. One of these subsequences was identified by Wade *et al.,* and ectopically introduced into the coding region of the *melA* gene. Significant *in vivo* binding was observed at this ectopic site [35]. As they share the CTG to ATG mutation, and are otherwise canonical LexA binding sites, the MotifCatcher-suggested sites shown in Table 1 are especially likely to bind LexA *in vivo*. The remaining 12 type III sites were also found to have degenerate subsequence matches (data not shown), however due to a lack of previously published data showing LexA binding to similar degenerate motifs, these matches were not directly interpretable. Interestingly, an additional MotifCatcher analysis carried out on only the 19 type III sites yielded a novel motif in 8 of the 19 sites (Additional file 1: Figure S4), however we were unable to discover a meaningful biological interpretation for this motif.

**Table 1:** Suggested degenerate LexA binding sites for unconventional LexA targets

**ChIP-chip score**	**Target gene(s)**	**Binding site coordinates**	**Binding site sequence**	**Mutation type**
1.78	ptrA	2957002:2957021	CTATGTTTATATAACCATCA	CTG->ATG x2
1.42	otsB,otsA	1980128:1980145	ATATGTGTTT-TA-CCATTG	CTG->ATG x2, 2del
1.41	yfaX,yfaW	2359315:2359334	ATATGATCGTCTATCCAGTG	CTG->ATG x1
1.30	ydjF,ydjK	1852887:1852906	CCCTGTATCTTTTTACATCA	CTG->ATG x1
1.03	ybeR,ybeS	676025:676044	AAATGTATTTAGGTACATGC	CTG->ATG x2
1.00	ynaE	1432307:1432326	ATATGTTGACTTATACATCG	CTG->ATG x2
0.77	trs5_1,mmuP	274515:274534	GGATGTTTAGATGTCCATAC	CTG->ATG x2

In the canonical LexA binding site consensus sequence, ATCTG(TA)_10_CAGTA, two ‘CTG’ half-site motifs in reverse compliment to each other separated by an intervening sequence of 10 nt are implicated as the most important sequence elements for protein-DNA contact. Among the MotifCatcher-suggested LexA binding site matches for unconventional (type III) sites, 7 of the 19 were defined by substituting one or both ‘CTG’ groups from the canonical consensus sequence with ‘ATG’ (1 of these 7 required an additional 2 nucleotide deletion mutation). The ChIP-chip score column (far left, taken from [35]) reflects the experimentally observed binding strength at each site. The *ptr* binding site sequence has been experimentally verified to bind LexA when inserted ectopically to a non-LexA-binding region [35]. Given that the other 6 sites in this list share the ‘CTG’ to ‘ATG’ mutation, it is likely that these MotifCatcher-suggested degenerate LexA binding sites also bind LexA *in vivo. *

#### Evidence for alternative Cse4p binding mechanisms in *S. cerivisiae*

We were interested to test MotifCatcher’s ability to characterize general binding profiles for proteins whose DNA binding signatures are longer and have fewer highly conserved elements than classical transcription factor binding site motifs. Cse4p is a histone H3 variant protein essential for kinetochore function in *S. cerevisiae*[39]. The protein is known to localize to the centromeric (CEN) site on each chromosome. The protein is recognized at these CEN sites by a ~125-bp stretch of three sequence elements (designated CDEI, CDEII, and CDEIII) [40]. A detailed analysis of the biophysical interactions of CEN-binding proteins suggests that Cse4p genetically interacts with only the CDEI and CDEII sites, with the majority of direct protein-DNA interaction occurring with the 74–86 bp A/T-rich CDE II segment [41]. A recent ChIP-seq experiment examining DNA-binding of Cse4p demonstrated that in addition to the 16 CEN binding sites, Cse4p also bound at 142 additional sites [42].

**Figure 5:**
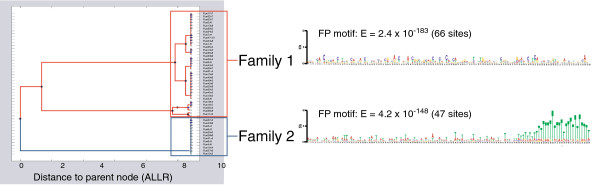
**Motif tree of ChIP-seq-derived Cse4p binding sites in Yeast.** Two distinct motif families were determined from the MotifCatcher analysis of 158 ChIP-seq derived Cse4p binding sites. Logos for the FPs of these two families taken with an FP frequency threshold of 1.0 are pictured above. At this threshold, all 158 sites naturally segregated into three non-intersecting groups (66 of the 158 into family 1, 47 of the 158 into family 2, and 45 of the 158 into neither family). While the associated motif in the first family does not resemble a readily recognizable sequence motif, the low E-value for this profile suggests that the position-specific distribution of sequence elements is highly different from the position-specific distribution of sequence elements in the whole Yeast genome. More than half of the sites in this family were also associated with highly ranked PolII targets, which supports the hypothesis put forth by [42] that Cse4p is transiently localizing to regions of high histone turnover. The A/T-richness of the second motif family resembles the canonical CDEII motif, which is known to be responsible for the majority of direct interactions between Cse4p and DNA.

We tested MotifCatcher’s ability to provide additional insight into the mechanism of Cse4p binding for these 142 non-centromeric sites. An iterative MEME/MAST MC search was carried out on a sequence data set derived from ChIP-seq derived coverage regions. The R_i_-associated motifs naturally clustered into two families (Figure 5). Familial profiles were generated for each family using an FP threshold of 1.0. A careful examination of the output revealed that the data set naturally segregated by motif into two non-overlapping groups of sequence entries. This separation of the data set by motif similarity suggests that from a sequence analysis point of view, this data set is more appropriately represented as two completely independent sets.

Lefrançois *et al*. determined a high correlation of the 142 novel Cse4p binding site target genes with the 100 highest ranked PolII targets (as determined by ChIP-seq [42]) finding 49 sites in common. This led to the hypothesis that the 142 novel binding sites were the result of transient localization of Cse4p to regions of high histone turnover. Our iterative MEME/MAST MC analysis segregated the data set into three non-intersecting groups: Family 1 (66/158 sites, all novel sites), Family 2 (47/158 sites, 16 CEN sites and 31 novel sites), and sites that belonged to neither group (45/158 sites, all novel). Of the 49 novel sites reported by Lefrançois et al. that associated with highly ranked PolII targets, 37/49 (71%) belonged to Family 1, 1/49 (2%) belonged to Family 2, and 11/49 (24%) belonged to neither group. The distribution of PolII target sites among unconventional sites in motif families 1, 2, or neither group could not be explained by a uniform normal distribution (*X*^2^, α = 0.001).

Our analysis suggests that sites included in Family 1, but not Family 2, may be consistent with Lefrançois *et. al’s* hypothesis of transient localization of Cse4p to regions of high histone turnover. While the motif defining Family 1 does not contain any highly conserved features (Figure 5), it does demonstrate a general sequence profile distinct from the 61.86% A/T content of the whole *S. cerevisiae* genome. The absence of sites in Family 2 in the list of the top 100 PolII sites suggests that this hypothesis is not appropriate for the sites in this family. However, the second motif family was defined by a A/T-rich motif similar to the canonical CDEII motif. All sixteen canonical CEN sites were found in this subset. Considering the similarity of the motif defining the second motif family to the canonical motif, we hypothesize that sites in this group describe sites where Cse4p interacts with DNA directly, in a manner similar to Cse4p binding to the CDEII region of canonical CEN sites.

Coordinating the motif families, familial profiles, and branching diagram outputs generated by MotifCatcher, we conclude that only a fraction (66/142) of the novel Cse4p binding sites previously thought to localize to sites of high histone turnover actually do so. Another fraction (31/142) of the novel sites report a sequence profile similar to the canonical CDEII motif, binding at these sites therefore likely occurs directly to the DNA following the binding mechanism associated with the CDEII region at centromeric sites.

#### Discovery of closely related motif variants for a family of homologous transcription factors: general transcription factors TfbB, TfbD, and TfbG in *Halobacterium* sp. NRC-1

One of the main mechanisms for the evolution of gene regulatory networks is the duplication and subsequent divergence of transcription factors [43]. Often this process happens multiple times and leads to the accumulation of families of transcription factors functioning simultaneously in the cell. Depending on the degree of divergence, multiple homologous transcription factors may target both overlapping sets of promoters and unique promoter sets. The resultant partitioning of gene regulatory networks by this mechanism is thought to be at the basis of some important functional properties of gene regulatory networks and could inform re-engineering of biological networks. At the root of this problem is the ability to distinguish functional differences between often-similar binding sites of homologous transcription factors.

In Archaea, two general transcription factors are thought to be necessary and sufficient for initiating basal transcription, homologs to eukaryotic (1) TFIIb (referred to in archaea as TFBs) and (2) TATA-binding proteins (TBPs) [44]. These general transcription factors are present in multiple copies in several archaea. For example, the genome of the archaeon *Halobacterium* sp. NRC-1 encodes 6 different TBP proteins and 7 different TFB proteins. With 6 different TBPs (*tbpA-F*) and 7 different TFBs (*tfbA-G*), *Hb. NRC-1* could use up to 42 different TBP-TFB complexes. Evidence for at least 7 of these has been observed [44]. In the same study, Facciotti *et al.* observed that the sets of genes bound by each of the TFB homologs in *Hb.* NRC-1 appear to be partially overlapping while still including distinct functional groupings [44]).

We hypothesized that given the overlap in promoter binding sites for many TFB assayed in [44], a binding motif common to all TFBs must be present and likely include a signature similar to the TFIIB binding element (BRE) described earlier [45,46]. We also proposed that direct protein-DNA interaction could play a role in discriminating between these different types of TFB binding sites. However, previous efforts to simply identify a TFB motif from ChIP-chip data using the MEME suite had been unsuccessful [44]. Testing this hypothesis requires (1) that there are subtle differences in the binding sites between these different TFB proteins, (2) an experimental protocol exists that could confidently reduce the search space for motif finding algorithms, and (3) a sensitive tool for detecting and classifying these differences can be applied. We investigated our binding hypothesis by using a new ChIP-seq protocol for *Hb.* NRC-1 [47] that provides greater spatial resolution than the previously adopted ChIP-chip [44] and MotifCatcher to help discover a general TFB motif, and if possible, distinguish subtle differences between the TfbB, TfbD, and TfbG binding sites.

**Figure 6:**
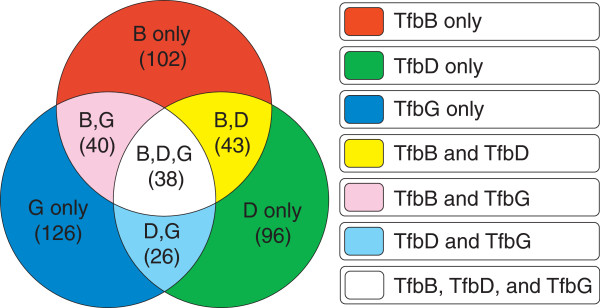
**Determination of 7 non-intersecting TFB binding site data sets.** Each colored section of the three-circle Venn diagram contains the number of ChIP-seq peak centers determined for each of three GTFs TfbB, TfbD, and TfbG. Peaks were resolved at a peak-to-peak center distance of 30 nt. Sequence entries were created by extracting a continuous block of sequence centered at each of the ChIP-seq peaks (each peak produces one sequence entry). Each of the colored segments of the diagram refers to a different data set of sequences. All determined peaks and corresponding sequence entries were limited to exist in only one of the 7 possible data sets.

Three separate ChIP-seq experiments were conducted as in Wilbanks *et al.*[47] for three strains of *Hb.* NRC-1, each encoding a chromosomally tagged copy of TfbB, TfbD or TfbG. Binding peaks were identified with the publicly available software package SPP [48] (sequence data available for download, NCBI accession number SRA048305.1). A comparison of peak lists generated for each ChIP-seq experiment revealed that among the putative binding sites, some were accessible to only one, some to any two, and some to all three of these TFB proteins. Using these criteria, we constructed 7 non-intersecting ChIP-seq peak lists, detailing the genomic locations of putative binding sites accessible to one, two, or all three TFB proteins (Figure 6).

For each of the 7 TFB-binding ChIP-seq peak lists, five groups of data sets of sequences were constructed: (1) 60-bp, (2) 100-bp, and (3) 200-bp stretches of sequence centered about the genomic location of each peak center, and (4) 60-bp stretches of sequence centered around a site displaced 60 nt upstream of the center of each ChIP-seq peak. We randomly shuffled the members of the 7 TFB-binding ChIP-seq peak lists into 7 new groups, and extracted 60-bp stretches of sequence centered about the genomic location of each peak center (5). In addition to these five groups, we created a final data set containing 126 random 60-bp stretches of sequence taken from around the *Hb.* NRC-1 genome.

Each data set was subjected to a MEME ZOOPS search, an iterative MEME/MAST MC search, a Gibbs recursive sampler search, and an iterative Gibbs sampler/MAST MC search. In selecting these four motif-finding strategies, we were able to compare the performance of two basic motif finders (the MEME Suite and recursive Gibbs sampler), with the performance of the MC iterative search extension of that finder. A particular statistically overrepresented sequence motif was repeatedly discovered from many of the Gibbs recursive sampler and MotifCatcher analyses (Additional file 2). In a few cases, a version of this motif was reported which included fewer than 5% of the input sequence entries, which we evaluated for performance statistics as a “half-discovery”. It was not always possible to discover the motif using a reasonably small number of seeds (100), however application of the MC iterative search program to an “ideal” seed (one built out of sequence entries known from other analyses to contain a version of the motif) could often yield re-discovery of the motif. Comparative statistics (Table 2) revealed that the MotifCatcher iterative search framework applied to both the MEME suite and Gibbs recursive sampler offered improved performance versus the motif finder by itself (the improvement was especially profound when MotifCatcher was applied to the MEME suite). If a much larger number of runs were carried out, one would expect the MotifCatcher program to randomly create an ideal or near-ideal seed, so the motif might now be discovered from data sets where 100 runs was insufficient for motif discovery.

**Table 2:** Ability to discover TFB motif from ChIP-seq data sets

**Motif-finding strategy**	**TP**	**TN**	**FP**	**FN**	**Sensitivity**	**Specificity**	**F-measure**
Theoretical best	28	8	0	0	1.00	1.00	1.00
MEME ZOOPS	5.5	8	0	22.5	0.20	1.00	0.33
Iterative MEME/MAST MC	16	8	0	12	0.57	1.00	0.73
Gibbs recursive	15	8	0	13	0.54	1.00	0.70
Iterative Gibbs/MAST MC	18	8	0	10	0.64	1	0.78
Iterative Gibbs/MAST MC +Ideal	23	8	0	5	0.82	1	0.90

Five groups of 7 sequence data sets were constructed from the putative binding sites derived from TfbB, TfbD, and TfbG ChIP-seq experiments. Various lengths of sequence taken surrounding each site (60bp, 100bp, and 200bp) were examined, as were stretches of sequence 60 base pairs long displaced a distance of 60 base pairs from ChIP-seq sites (displaced), and data sets built from randomly shuffling binding sites from the 7 different TFB binding site groups into new groups of equal size (shuffled). A data set of 126 60-bp segments from the *Hb* sp. NRC-1 genome was generated as an additional control (random). Evaluation of these 36 data sets with 4 alternative motif-finding strategies revealed distinct differences in the ability of each strategy to discover the putative TFB motif. For all data sets except the random and displaced data sets, discovery of a strong match to the TFB motif was scored as a true positive (TP). Failure to discover a TFB motif could from the random and displaced datasets was scored as a true negative (TN). If a weak match to the TFB motif was discovered in any dataset other than a random or displaced data set, it was scored as a half TP (0.5). One hundred runs were carried out for the Iterative MEME/MAST MC and Iterative Gibbs/MAST MC runs. A number of ‘ideal’ seeds were artificially created and were found to converge to the TFB motif. Given a very large number of runs, an ideal seed or near-ideal seed is expected to occur by chance, so the TFB motif would be recovered in these cases. For both MEME and the Gibbs recursive sampler, the application of the MotifCatcher extension significantly improved each finder’s performance.

**Figure 7:**
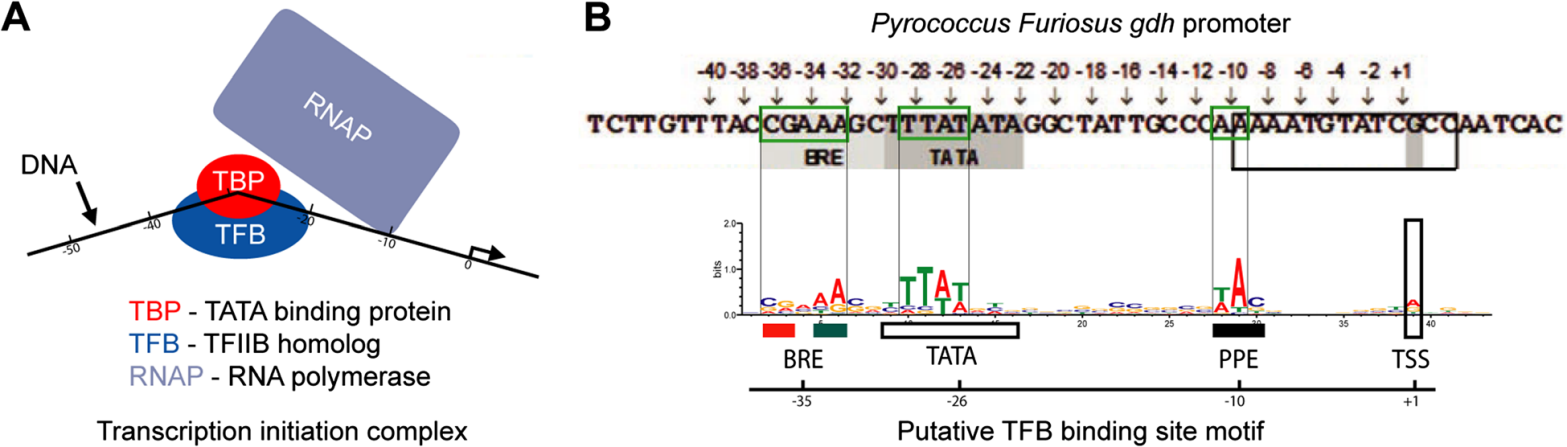
**TFB and TBP binding to DNA in Archaea.** (**A**) Illustration of coordinated binding of TBP/TFB to form a transcription initiation complex on DNA. Note significant bending of DNA around bound TBP/TFB complex. (**B**) Reproduction of *P. furiosus gdh* promoter sequence [49] (top) compared to logo alignment of MotifCatcher-derived motif (bottom). In the *gdh* promoter sequence, shading indicates the TATA box and BRE elements and a black rectangle indicates the transcription-bubble region. Positions above the sequence are relative to the TSS, and arrows indicate phosphates analyzed in [49]. The logo motif was formed by aligning sequences near 223 of the 417 (53%) reported TfbB, TfbD, and TfbG ChIP-seq peaks. Conserved elements between the *gdh* promoter and MotifCatcher-derived motif are indicated by green boxes surrounding the *gdh* promoter elements and by black lines extending vertically from the motif to *gdh* promoter sequence. The putative BRE, TATA box, PPE, and TSS in the logo are indicated with colored rectangles (red and green, BRE; white, TATA box; PPE, black; TSS, vertical white), and text annotation. An axis under the motif displays the most commonly discovered distance of sequence elements relative to the closest TSS in the *Hb.* NRC-1 genome. The distance of elements from the *Hb.* NRC-1 motif logo and elements in the *P. furiosus gdh* promoter to their respective TSS matched precisely.

In Archaea, TBP binds to the 8bp A/T rich TATA box sequence. Localization of a TBP to DNA forms a bent protein-DNA complex, which is recognized by a TFB. The TFB binds to both the TBP through protein-protein interactions, and to DNA at the adjacent BRE (Figure 7A). The TFB/TBP forms a transcription initiation complex, which then binds RNA polymerase (RNAP). A DNase footprinting assay applied to the *gdh* promoter in the hyperthermophilic marine archaeon *Pyrococcus furiosus* revealed that in addition to the contacts upstream of the TBP protein at the BRE, TFB makes significant contacts downstream, approaching the transcript start site (TSS) [49]. An alignment of our discovered motif to the *P. furiosus gdh* promoter region (Figure 7B) revealed a precise mapping to the putative BRE, TATA box, and PPE (proximal promoter element) sites. The PPE, which in general comprises any A/T-rich sequence elements between −12 to −1 relative to the transcription start site, has been implicated as essential to the activity of the 16S/23S rRNA promoter of the archaeon *Sulfolobus shibatae*[50]. This sequence may be essential for stabilizing contacts between the N-terminus of TFB and RNAP [51]. Examination of the promoters in several halophilic archaea has revealed a statistically overrepresented A/T rich pattern located precisely at −10 relative to the TSS [52,53], which is in exact agreement with the location of the putative PPE. In the majority of cases, the distance between elements in our discovered motif to ATG-predicted and experimentally determined TSS in *Hb.* NRC-1 agreed exactly or nearly exactly with corresponding elements in the *P. furiosus gdh* promoter (Additional file 2)*.*

**Figure 8:**
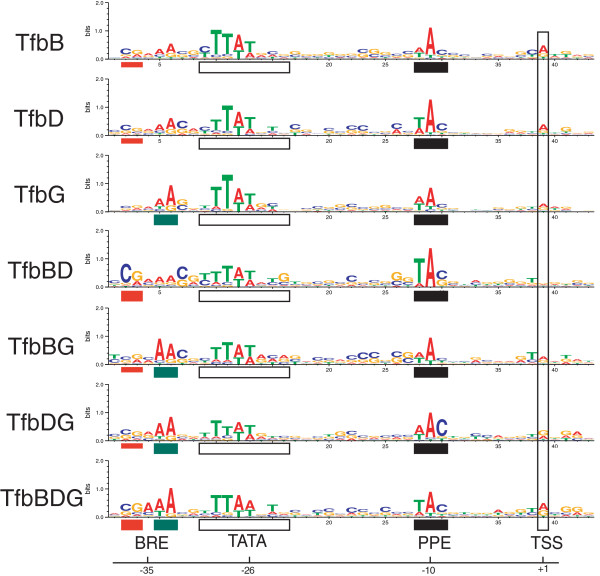
**Differences among TFB motifs among sequence data sets.** A multiple alignment of significant matches to the TFB motif from each data set reveals the general BRE-TATAbox-PPE-TTS layout for all data set. The putative BRE, TATA box, PPE, and TSS in the logo are indicated with colored rectangles (red and green, BRE; white, TATA box; PPE, black; TSS, vertical white), and text annotation. Differentially emphasized elements within the BRE may confer specificity for binding of one or more TFB proteins. Specifically, an emphasized ‘AA’ 4 bp upstream of the TATA box was discovered in all datasets that contain sites that bind TfbG (turquoise box, TfbG, TfbBG, TfbDG, and TfbBDG), and ‘CG’ 7bp upstream of the TATA box was emphasized most for sites that can bind both TfbB and TfbD (full red box, TfbBD and TfbBDG data sets), intermediately for sites that can bind either TfbB or TfbD but not both (half red box, TfbB, TfbD, TfbBG, TfbDG data sets) and least for sites that can bind neither TfbB nor TfbD (TfbG). The similarities and differences discovered within the BRE invite future experimental investigations.

A multiple alignment of sequence entries from each individual data set was constructed which revealed differentially emphasized elements in the putative BRE component (Figure 8). The variation among conserved sequence elements within the BRE invites several hypotheses regarding DNA-sequence specificity for binding one or more of the tested TFB proteins: Sites that could bind TfbG (TfbG, TfbBG, TfbDG, and TfbBDG) all contain a pronounced ‘AA’ 4bp upstream of the TATA box. Sites that do not bind TfbG do not show this pronounced ‘AA’. A ‘CG’ pattern located 7 bp upstream of the TATA box is most pronounced in sites that can bind both TfbB and TfbD (TfbBD and TfbBDG), intermediately pronounced in sites that bind either TfbB or TfbD but not both (TfbB, TfbD, TfbBG, TfbDG) and significantly less pronounced in sites that cannot bind either TfbB or TfbD (TfbG). A sequence analysis of all TFB proteins in *Hb.* NRC-1 demonstrated that the TFBs naturally segregated into four distinct groups, one of which contained both TfbB and TfbD, another contained TfbG [47]. The variation among the protein structures might be recapitulated in variation among the protein-DNA binding sites.

## Conclusions

The MotifCatcher algorithm frames an existing pattern detection tool (motif finder) in a Monte Carlo simulation framework, and organizes significant candidate output motifs produced by that tool into a branching diagram that may be further processed. It is not in itself a motif-finder, but rather a generic strategy to extend the sensitivity and utility of existing motif-finders. Here, we demonstrated that application of two motif-finders supplemented with our MotifCatcher extension algorithm resulted in an increase in performance compared to the motif finder alone (LexA binding in *E. Coli* and TFB binding in *Hb.* NRC-1). We demonstrated this result using two widely used motif finders that rely on different motif-finding strategies (the MEME Suite and recursive Gibbs sampler).

Beyond simple increases in detection performance, our novel approach of organization of candidate motifs in a tree diagram highlighted an exciting feature of MotifCatcher: In our analysis of ChIP-seq-derived Cse4p binding in yeast, we discovered that the input sequence entries naturally segregated into two non-intersecting groups, entirely according to the discovered motifs. We were able to support the conclusion that this segregation is appropriate by discovering a very similar segregation of the data set based on correlation with polII binding sites. From our motif-finding analyses related to homologous TFBs in *Hb.* NRC-1, we were able to repeatedly discover a statistically significant motif present in all of the data sets. Comparing this motif with an archaeal promoter sequence used in a DNase footprinting experiment, we were able to map significant elements of our motif to the well-characterized TATA box, BRE, and PPE motifs. A comparison of the distance of elements in our motif to annotated and experimentally determined transcript start sites was in precise agreement with the distances described from the DNase footprinting experiment. Significant differences were discovered among the BRE element as it was discovered from the different TFB datasets. We hypothesize that these differences confer specificity for binding among various TFB proteins. Our results suggest an experimental investigation, which could further elucidate crucial details of the mechanisms of TFB binding in *Hb.* NRC-1.

In general, MotifCatcher may be used to suggest ways that a single data set might be more appropriately segregated into several smaller data sets (as demonstrated in both the *S. cerevisiae* and *Hb.* sp. NRC-1 analyses). The MotifMap utility, which maps discovered motifs to the input sequence entries themselves, coupled with statistical measures to evaluate significant co-occurrences and co-localizations of significant motifs, increases the power of this feature. In addition to improving motif detection sensitivity, MotifCatcher allows one to better organize and categorize biological sequence datasets based on discovered motifs.

## Methods

### Algorithm details

All desired specifications regarding the nature of the sought motif (minimum width, maximum width, the option to check for motif instances on the reverse compliment strand, the option to force the motif to be palindromic in nature, etc.), are user-input. These specifications are applied to the motif-finding search, which in this implementation is accomplished by the MEME ZOOPS (Zero or One Occurrence Per Site) model. For motif searches, a background model can either be supplied by the user or built from $\bar{Y}$ (with order appropriate for the total number of characters in $\bar{Y}$).

In the MotifCatcher software package, a GUI interface allows the user to navigate the consequences of segmenting a motif tree at various clustering thresholds. The clustering threshold varies according to the topology of the tree, but as a general rule, the clustering threshold should be quite stringent (only highly similar R_i_-associated motifs are grouped together). This preference is incorporated into the MotifCatcher software default settings.

### Software implementation

The MotifCatcher software platform in its current implementation in wide release coordinates with (1) the MEME suite (v. 4.5.0), and (2) the STAMP platform (v. 1.1). Both programs must be installed and configured correctly prior to MotifCatcher installation. MotifCatcher is implemented in MATLAB, and beyond standard MATLAB toolboxes, relies on MATLAB’s commercially available (1) bioinformatics toolbox.

The MotifCatcher software is freely available at the Facciotti lab website ( http://www.bme.ucdavis.edu/facciotti/resources_data/software/).

### Preparation of biological data

The MEME Suite version 4.5.0 (including the MAST program, version 4.5.0) was used for all MEME searches and MC iterative MEME/MAST searches, and the Gibbs recursive sampler version 3.1 was used for all Gibbs searches and MC iterative Gibbs/MAST searches. For all motif searches, motifs could be discovered on the forward or reverse strand, with no preference to discover palindromic motifs. A 3^rd^-order background model was generated from all sequences in $\bar{Y}$ and applied to all MEME and MotifCatcher MEME searches (unless otherwise mentioned). Sequence entries were set to have 0 or 1 instances of a motif. For all MotifCatcher searches, whenever MAST was incorporated in a related subset determination protocol, it was always used with its default similarity threshold value of 10. All $R_{i}\subset \bar{R}$ with an associated motif with an E-value greater than 0.001 were not included in the construction of the motif tree, and were excluded from further analyses. Motif trees were built by comparing the ALLR (average log-likelihood ratio) [23] between R_i_-associated motifs, and a distance tree was constructed using UPGMA linkage. All R_i_ in the motif tree were segregated into families using a clustering threshold of 5% of the maximum dissimilarity discovered among the whole set of all R_i_-associated motifs. All motif logos were created using WebLogo version 3.1 [54].

### LexA binding in *E. coli*

Relevant sequence data sets were created by extracting 1000-bp regions centered at the reported ChIP-chip peaks reported by *Wade et al.* The original data set consisted of the original 1000-mers from the ChIP-chip experiment (type I, II, and III sites). The random substituted data set was composed of the original type I and type II 1000-mers with 19 non-overlapping 1000mers from random genome sites. All motif searches utilized the standard set of motif-finding parameters, where motifs could be anywhere from 10 to 30 nucleotides in length. The number of seeds created for each MC motif search varied to generate comparable motif trees for each related subset determination option (500, 200, and 50 seeds, respectively, for the MEME ZOOPS MC search, single MAST MC search, and iterative MEME/MAST MC search). Aside from the disparate number of seeds, all MC motif searches were identical.

### Evidence for alternative Cse4p binding mechanisms in *S. cerevisiae*

ChIP-seq enrichment regions were taken from supplemental data by Lefrancois *et al.*[42]. Accounting for 3 replicate experiments, there were 158 sequence entries, with 16 large peaks associated with the CEN regions, and 142 smaller peaks corresponding to novel Cse4p binding sites. An iterative MC MEME/MAST search was undertaken with 50 random seeds containing 8 sequence entries, searching for motifs of 125 bp in length (the length of the canonical CDEI-CDEII-CDEIII motif). A 3^rd^-order background model was generated from the whole genome (strain S288c) and applied to all motif searches.

Nearby open reading frames for the 100 highest-scoring polII sites, based on the number of excess reads (sample reads – input reads) as determined from the first replicate experiment performed by *Lefrancois et al.*[42], were extracted and compared to the closest open reading frame to the binding sites of the Cse4p protein (as determined by ChIP-seq experiment). The MotifCatcher-derived family was determined for each of the 49 sites (1/49 from Family 1, 37/49 from Family 2, and 11/49 in neither group). Based on this breakdown, the null hypothesis that the 49 sties were randomly distributed over the 3 MotifCatcher-derived families had to be rejected at a confidence of *α* = 0.001._._

### Discovery of closely related motif variants for a family of homologous transcription factors: general transcription factors TfbB, TfbD, and TfbG in *Halobacterium* sp. NRC-1

In the determination of genomic sites accessible to one or more TFB proteins, peak centers within 30 nt of each other were considered to be the same site (in accordance to the resolution of the ChIP-seq experiment [47]). In all motif searches, motifs could be anywhere from 6 to 30 nt in length. For iterative MC MEME/MAST runs, 10% of the sequence entries in the whole input data set were selected for each random seed, for iterative MC Gibbs sampler/MAST runs, seeds always consisted of 20 randomly selected sequence entries.

For evaluating the results of motif finding applied to TFB sequence data sets, all statistically significant output motifs were manually evaluated. Only motifs containing sequence elements resembling the TATA box, BRE, and PPE sequence motifs at the appropriate spacing were considered TFB motif matches. In the multiple alignments of representative TFB motifs, a sequence data set of 100 nt regions centered at all discovered peaks was constructed, and scanned with MAST for the putative TFB motif with a p-value threshold of 0.01 and an E-value threshold of 100. Sites that showed multiple matches, or matches far from the center of the sequence file were discarded. All remaining matches were aligned, with 9 additional bp at the 3’ end added in the alignment. Logos were constructed using weblogo (v.3.1). 233/417 (53%) of TFB binding sites were used in the alignment - Specifically, 51/102 (50%) from TfbB; 47/96 (49%) from TfbD; 51/126 (41%) from TfbG; 22/43 (51%) from TfbBD; 20/40 from TfbBG (50%); 19/26 from TfbDG (73%), and 23/38 (61%) from TfbBDG.

## Abbreviations

ChIP: Chromatin immunoprecipitation; GTF: General transcription factor; ChIP-chip: Chromatin immunoprecitation with hybridization by microarray; ChIP-seq: Chromatin immunoprecipitation with massively parallel sequencing; MEME: Multiple expectation maximization for motif elicitation; MAST: Motif annotation search tool; ZOOPS: Zero or on occurrence per site; OOPS: One occurrence per site; MC: MotifCatcher; STAMP: Similarity, tree-building, and alignment of DNA motifs and profiles; UPGMA: Unweighted paired group method; SOTA: Self-organization tree algorithm; FP: Familial profile; Hb. NRC-1: *Halobacterium* sp. NRC-1; TFB: Transcription factor IIb homologue; TBP: TATA-binding protein; RNAP: RNA polymerase; BRE: TFB recognition element; PPE: Proximal promoter element.

## Competing interests

The authors declare that they have no competing interests.

## Authors’ contributions

PS developed and implemented the MotifCatcher algorithm, applied MotifCatcher to all data sets, interpreted the results, and drafted the manuscript. EGW helped carry out all ChIP-seq experiments, supplied binding site coordinate data to PS, and helped revise the manuscript. DL helped carry out all ChIP-seq experiments. MTF Oversaw the analyses, assisted the interpretation of results, and helped revise the manuscript. All authors read and approved the final manuscript.

## Funding

This work was funded by NSF grant #EF0949453 and startup funds to MTF.

## Supplementary Material

Additional file 1 **Figure S1.** Gcn4 Retrieval as a function of dataset corruption. **S2.** Alternative extended description of the MotifCatcher algorithm. Monte Carlo framework. Motif tree construction. Organization and evaluation. Software platform. **S3.Figure S2.** Screenshots of MotifCatcher software platform. S4: **Figure S3**. Motif Finder performance on LexA data set as a function of FP frequency threshold. **S5.** Supplementary Excel Spread Sheet. Table of Contents. Recovery of LexA motif with variable FP Threshold. Ability for various motif finders to discover TFB motif. Comparison of MotifCatcher-discovered motifs with TSS. **S6.Figure S4.** Novel motif discovered in Type III LexA binding sites. Supplementary References. Click here for file

Additional file 2 Recovery of LexA motif with variable FP threshold, Comparison of the ability of various motif finders to discover the TFB motif, and comparison of MotifCatcher-discovered motifs with location of experimentally determined Transcript Start Sites. Click here for file
