# Supplementary material for: A Monte Carlo-based framework enhances the discovery and interpretation of regulatory sequence motifs
Source: BMC Bioinformatics. 2012 Nov 27;13:317. doi: 10.1186/1471-2105-13-317 (PMC3542263; doi:10.1186/1471-2105-13-317)
Supplement: Additional file 1 — Figure S1. Gcn4 Retrieval as a function of dataset corruption. S2. Alternative extended description of the MotifCatcher algorithm. Monte Carlo framework. Motif tree construction. Organization and evaluation. Software platform. S3.Figure S2. Screenshots of MotifCatcher software platform. S4: Figure S3. Motif Finder performance on LexA data set as a function of FP frequency threshold. S5. Supplementary Excel Spread Sheet. Table of Contents. Recovery of LexA motif with variable FP Threshold. Ability for various motif finders to discover TFB motif. Comparison of MotifCatcher-discovered motifs with TSS. S6.Figure S4. Novel motif discovered in Type III LexA binding sites. Supplementary References. [file 1471-2105-13-317-S1.docx]

**Supplementary Information**

Table of Contents

S1: Supplementary Figure 12

Gcn4 Retrieval as a function of dataset corruption2

S2: Alternative extended description of the MotifCatcher algorithm4

Monte Carlo framework4

Motif tree construction5

Organization and evaluation6

Software platform7

S3: Supplementary Figure 211

Screenshots of MotifCatcher software platform11

S4: Supplementary Figure 313

Motif Finder performance on LexA data set as a function of FP frequency threshold13

S5: Supplementary Excel Spread Sheet15

Table of ContentsSheet 0

Recovery of LexA motif with variable FP ThresholdSheet 1

Ability for various motif finders to discover TFB motifSheet 2

Comparison of MotifCatcher-discovered motifs with TSSSheet 3

S6: Supplementary Figure 416

Novel motif discovered in Type III LexA binding sites16

Supplementary References17


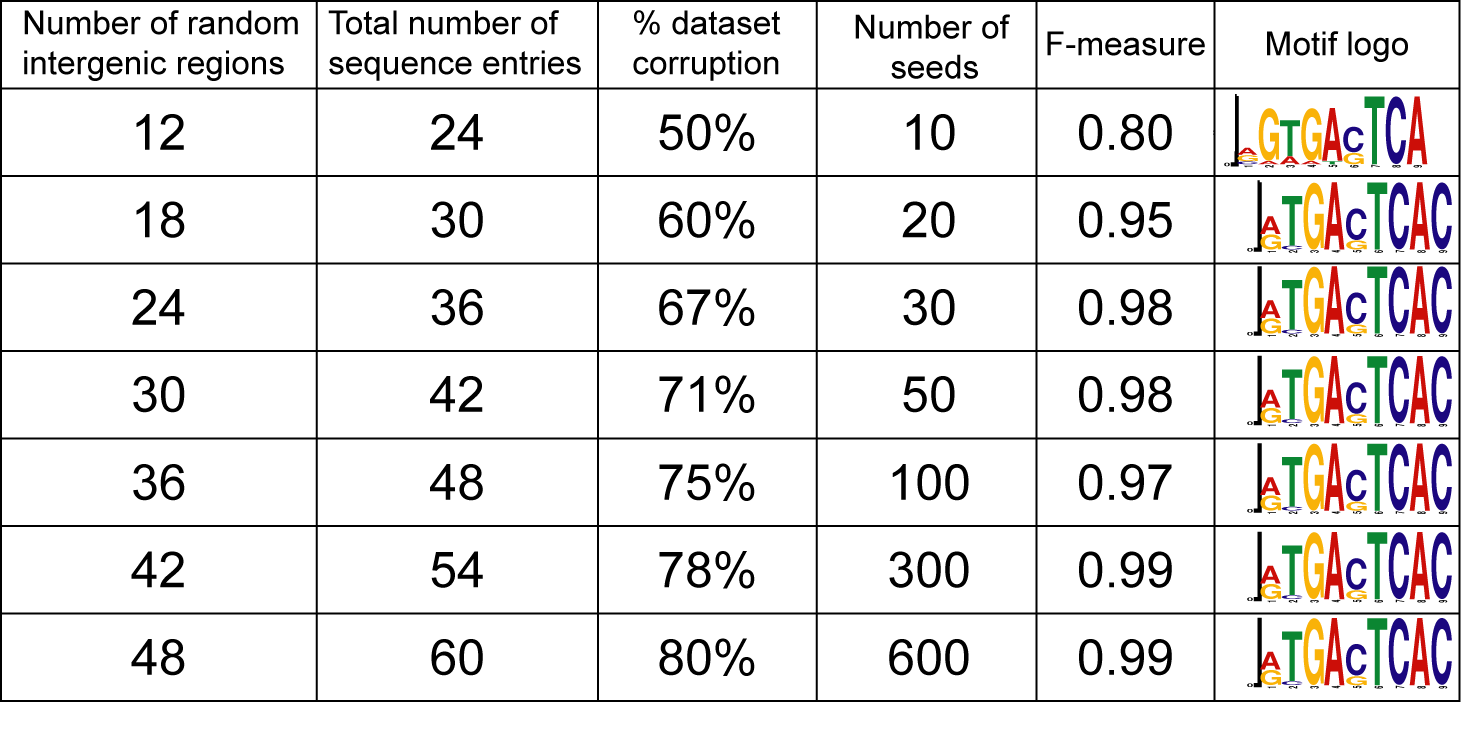


**Supplementary Figure 1: Gcn4 motif retrieval as a function of dataset corruption.** Gcn4 is a well-studied transcriptional activator protein associated with the amino acid control depression response in *Saccharomyces cerevisiae*. The canonical Gcn4 binding site motif [S1] spans 9 bp in length. From a genome-wide location analysis performed on *Saccharomyces cerevisiae* [S2] and subsequent re-analysis to more accurately identify direct and indirect transcription-factor DNA interactions [S3], a subset of 12 of the more than 7,000 intergenic regions from the *Saccharomyces cerevisiae* (yeast) genome were expected to bind directly to the transcription factor Gcn4 with high probability.

In our investigation, starting with the 12 intergenic regions contain an experimentally confirmed Gcn4 binding site and corresponding motif, we systematically added random, non-Gcn4-binding-site containing intergenic regions to the input data set (thus “corrupting” the input set with non-motif-containing sequence entries), and carried out motif searches. Motifs searches were carried out with a length specification of 9 bp in length (the length of the canonical Gcn4 motif). We discovered that MEME failed to recover the canonical Gcn4 motif when 12 additional random non-motif-containing intergenic regions were added to the data set (data not shown). MotifCatcher was able to recover the canonical Gcn4 motif comfortably when up to 48 of additional random non-motif-containing intergenic regions were added to the data set. The motifs were discovered with near-perfect sensitivity and specificity (above).

**Alternative extended description of the MotifCatcher algorithm**

Please refer to **Fig. 1** in the main text for a flowchart of the MotifCatcher algorithm. The following describes an implementation specifically tailored for use with the MEME Suite, though in principal any motif-prediction algorithm could replace MEME, and any motif-scanning algorithm could replace MEME Suite component program MAST (Motif Annotation Search Tool) [23]. The MEME Suite was chosen based on its confirmed effectiveness, usability, and prevalence in the bioinformatics community.

***Monte Carlo framework***

From an input data set of N sequence entries
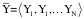
, n random seed subsets
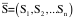
 are extracted, where each
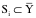
. ‘n’ is a user-specified value, and should be selected according to the size of
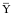
 and the expected number of
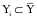
 that are thought to contain a subsequence instance of a significant motif (please see ‘Results’ section in the main text for examples). All desired specifications regarding the nature of the motif (minimum width, maximum width, the option to check for motif instances on the reverse compliment strand, the option to force the motif to be palindromic in nature, etc.), are user-input, and applied to the search at the point of utilization of actual motif-finding, which in this implementation is accomplished by the MEME ZOOPS (Zero or One Occurrence Per Site) model. In motif searches, a background model based on the individual frequencies of all possible single nucleotide to n-mer combinations of nucleotides is implemented, either supplied by the user, or built from
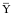
 (with order appropriate for the total number of characters in
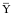
). Three alternative schemes are available to create a library of related subsets
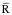
 from the set of seed subsets
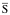
, applied to each
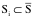
: (1) MEME ZOOPS MC (MotifCatcher) search: A MEME ZOOPS search is applied to S_i_, and sequence entries that contain subsequences included in the construction of the MEME ZOOPS-produced motif comprise R_i_. (2) Single MAST MC (MotifCatcher) search: A MEME ZOOPS search is applied to S_i_, and the MEME ZOOPS-produced motif is scanned over
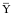
 using the MAST. All sequence entries in
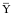
 that contain a significant subsequence match to the preliminary motif comprise R_i_. (3) Iterative MEME/MAST MC (MotifCatcher) search: A MEME ZOOPS search is applied to S_i_, and the MEME ZOOPS-produced motif, M, is scanned over
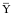
 using the MAST. All sequence entries in
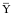
 that contain a significant subsequence match to the preliminary motif (M) comprise a modified seed
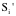
. MEME ZOOPS is applied to the modified seed
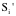
, which produces a modified motif, M’. M’ is scanned over
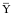
 using the MAST as before, and repeated MEME and MAST searches continue until convergence: a MEME search of a modified seed
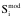
 produces a motif
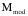
, and a MAST search of
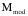
 over
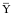
 finds subsequence instances of
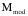
 in (and only in)
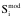
. The sequence entries in
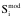
 comprise R_i_.

***Motif tree construction***

A branching diagram is constructed comparing the relative similarity of motifs associated with each of the different related subsets R_i_ in
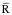
(motif tree). Some of the R_i_-associated motifs are likely not to be statistically significant, so all
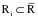
 with an R_i_-associated motif with an E-value higher than a user-specified maximal E-value threshold (typically, this value should be no larger than 0.01) are excluded from further analysis. As
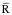
 may be very large, All R_i_ except a small subset of
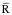
 with the lowest R­_i_-associated motif E-values may be excluded. The STAMP platform (Similarity, Tree-building, and Alignment of DNA Motifs and Profiles) [44,45] is utilized to organize the remaining
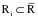
into a distance tree according to similarities of their R_i_-associated motifs. The pair-wise distance between two motifs is computed in a column-by-column fashion, using one of the following statistical metrics (selected by the user): Pearson correlation coefficient (PCC), Chi-square distance (pCS), average Kullback-Leibler divergence (AKL), sum of squared distances (SSD), or average log-likelihood ratio with or without a lower limit (ALLR or ALLR_LL). After a similarity distance has been computed between every R_i_-associated motif and every other R_i_-associated motif, the distance tree is assembled using an un-weighted paired group method (UPGMA) or self-organizing tree algorithm (SOTA). For a complete description of these methods, please refer to [44].

***Organization and evaluation***

The
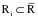
 represented in the motif tree will naturally cluster into groups according to the similarity of their R_i_-associated motifs. Each motif family F_i_ is a collection of R_i_ (grouped by similarity among their R_i_-associated motifs). The motif tree is therefore defined by a set of m non-intersecting motif families
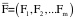
. The division of the motif tree into a set of non-intersecting motif families requires that a clustering threshold be imposed upon the
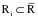
 represented in the tree. This clustering threshold varies according to the topology of the tree, and so in the MotifCatcher software package, a GUI interface allows the user to navigate the consequences of segmenting a motif tree at various clustering thresholds. As a general rule, the clustering threshold should be quite stringent (only highly similar R_i_-associated motifs are grouped together). This preference is incorporated into the MotifCatcher software default settings. Each motif family F_k_ is a collection of R_i_, and each R_i_ is a collection of sequence entries taken from the whole input set
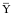
. The set of motif families
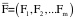
 is determined based entirely on the similarity of R_i_-associated motifs, without regard to the sequence entries from which subsequences are drawn to create these R_i_-associated motifs. There is value in describing a particular motif family F_k_ not just in terms of the collection of R_i_ that comprise F_k_, but also in terms of a singular characteristic motif, a familial profile (FP). Among the collection of R_i_ that form F_k_, some sequence entries will be re-discovered frequently among the R_i_, and some more infrequently. Subsequences drawn from sequence entries common to many R_i_ in F_k_ build more of the R_i_-associated motifs, and so are more representative of a general motif character of the family. An FP is generated for each F_k_ according to a user-selected FP frequency threshold: Y_j_ featured among R_i_ in all
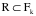
with a frequency of greater than or equal to this threshold are collected, and a motif is extracted from only these sequence entries using a MEME OOPS (one occurrence per site) model. The FP is the most significant motif representation of that family, and the subset from which the FP is built the most significant related subset.

**Software Platform**

The MotifCatcher software platform in its current publically available implementation coordinates with (1) the MEME suite, and (2) the STAMP platform. Both programs must be installed and configured correctly prior to MotifCatcher installation. MotifCatcher is implemented in MATLAB, and beyond standard MATLAB toolboxes, relies on MATLAB’s commercially available (1) bioinformatics toolbox, (2) symbolic toolbox, and (3) the SetPartFolder introduced and implemented by Bruno Luong (freely available on the MATLAB file exchange [www.themathworks.com/FES/efssfdfd](http://www.themathworks.com/FES/efssfdfd).). The MotifCatcher software is freely available at the Facciotti lab website (<http://www.bme.ucdavis.edu/facciotti/resources_data/software/>) and the MATLAB public file exchange (<http://www.mathworks.com/matlabcentral/fileexchange/32100>).

After invocation of the MotifCatcher program (which yields the start window depicted in **Supplemental Fig. 2a**), the user may choose to run a complete analysis on an input data set (producing a set of related subsets
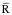
, a motif tree of significant
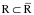
, families from the motif tree as determined by an input clustering threshold, and an FP for each family F_k_). Using this option demands that the user define all parameters initially. Parameters are organized according to the steps of the MotifCatcher algorithm they affect, and useful defaults are suggested. Instead of analyzing a data set from start to finish, the user may also choose to complete any single step in the MotifCatcher analysis – following completion of this step, the user is queried if they would like to continue to the next step in the pipeline, using the parameters and results generated by the previous step.

The point of segregating a motif tree into families (non-intersecting collections of related subsets) and generating familial profiles (FPs) for these families is a significant one in the analysis. The user may wish to perform this step many times at many different clustering and FP frequency thresholds. To facilitate easy re-computation of
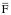
 and corresponding FPs, a GUI interface has been developed for this step (**Supplemental Fig. 2b**). Each individual R_i_-associated motif within a family may be thought of as an approximation of a ‘true motif’. Aggregating many similar R_i_-associated motifs produces a combination of a true motif with extraneous noise. Specifying a strict FP frequency threshold produces a conservative estimate of the true motif, and the sequence entries that contribute a subsequence to this motif. Specifying a more lenient FP frequency threshold produces a more liberal estimate of the true motif, incorporating less-frequently selected subsequences into the FP. Especially for cases where the motif is fundamentally degenerate, there may be great value in recreating the FP with more or fewer subsequences. In these cases, it may not be clear at what degree of degeneracy a subsequence should be included or excluded from the FP computation – including more subsequences in the FP computation will tend to include more false positives (random noise imitates a biologically significant subsequence), while including fewer sequences in the FP computation will include more false negatives (more distant biologically significant subsequences are not included, and not incorporated in the computation of the motif).

If a MotifCatcher-output R_i_ is small relative to
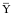
, this could mean that (1)
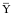
 is highly corrupt or (2) the motif determined for that particular R_i_ is not the central motif defining
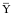
, but rather a secondary motif. The second case is more likely, and highlights a utility of MotifCatcher: Small subsets of
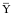
, which may contain motifs ‘hidden’ from view by motifs in a large portion of the Y_i_ in
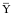
, may now be discovered. However, as R_i_ becomes smaller relative to
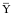
, and the motif associated with R_i_ becomes shorter and/or more degenerate, it becomes progressively more likely to detect the motif purely by random chance. Small MotifCatcher-suggested R_i_ and relative to
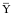
 and associated motifs should always be examined carefully. The motif map utility, which compares the localization of motifs in sequence entries across the whole data set
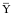
, may be helpful in sifting out real motifs from statistical artifacts.

After every major step in the MotifCatcher pipeline has been a completed, an output folder is created, and a MATLAB structure titled ‘DataSetProfile.mat’ is created and automatically saved to that folder with all relevant input and output variables. This makes it easy to investigate results at any point in the pipeline, simply by loading the DataSetProfile structure in the MATLAB window.
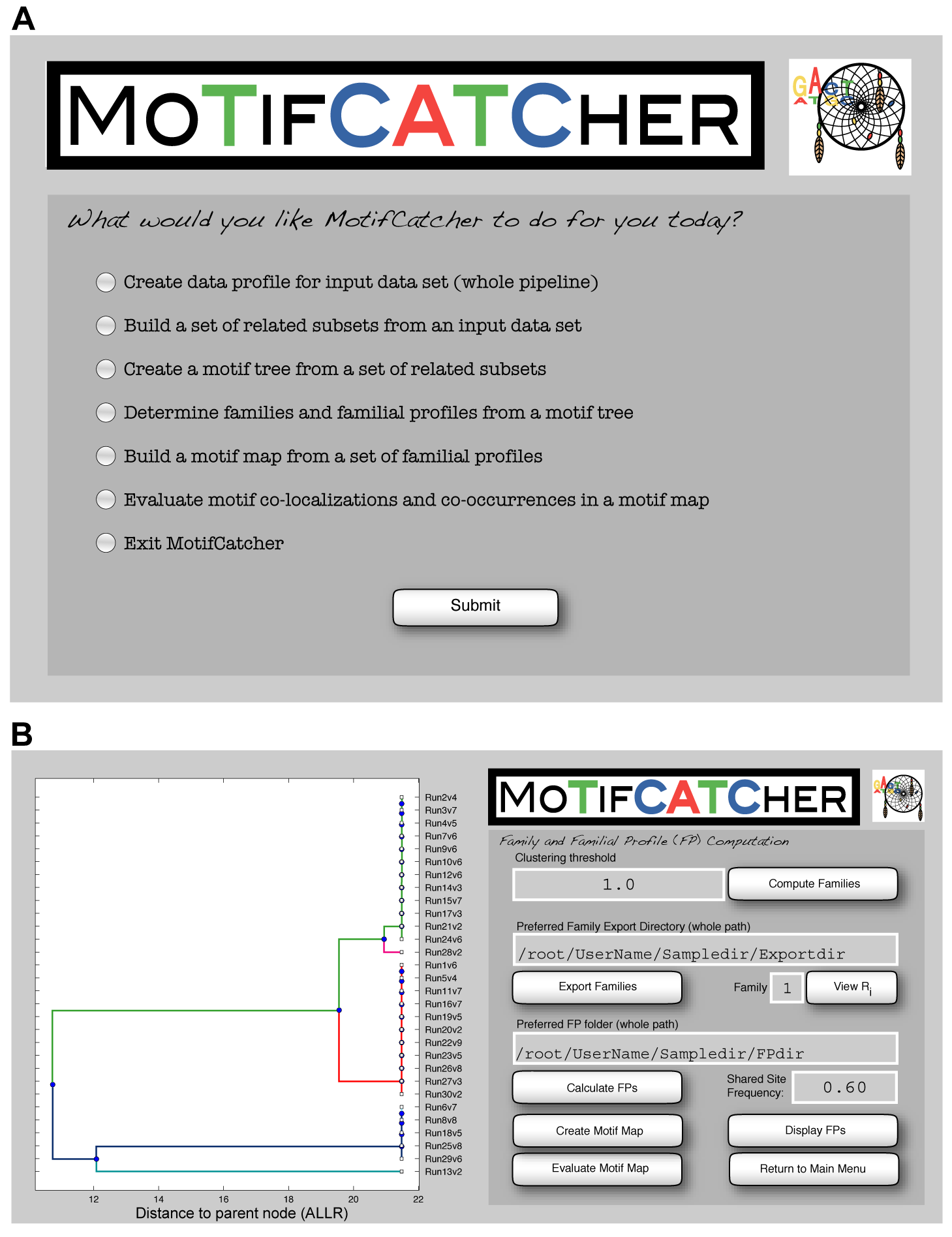
 **Supplementary Figure 2. Screenshots of MotifCatcher software platform**.

MotifCatcher’s intuitive GUI interface increases it’s accessibility. (A) The user may choose to carry out a complete, comprehensive analysis of an input data set of sequences, or complete any one of the four major steps in the pipeline (build a set of related subsets, create a motif tree, determine families and familial profiles, or create a motif map). The user may also evaluate a motif map for co-localizations and co-occurrences, or exit the program. (B) Motif trees are rendered in an interactive, GUI interface upon selection of the ‘Determine families and familial profiles’ step. The user may re-analyze the same motif tree several times using several different sets of parameters, with all analyses automatically saved and exported to desired locations. Related subsets clustered into a common family are indicated in the tree viewer window by colored branches.


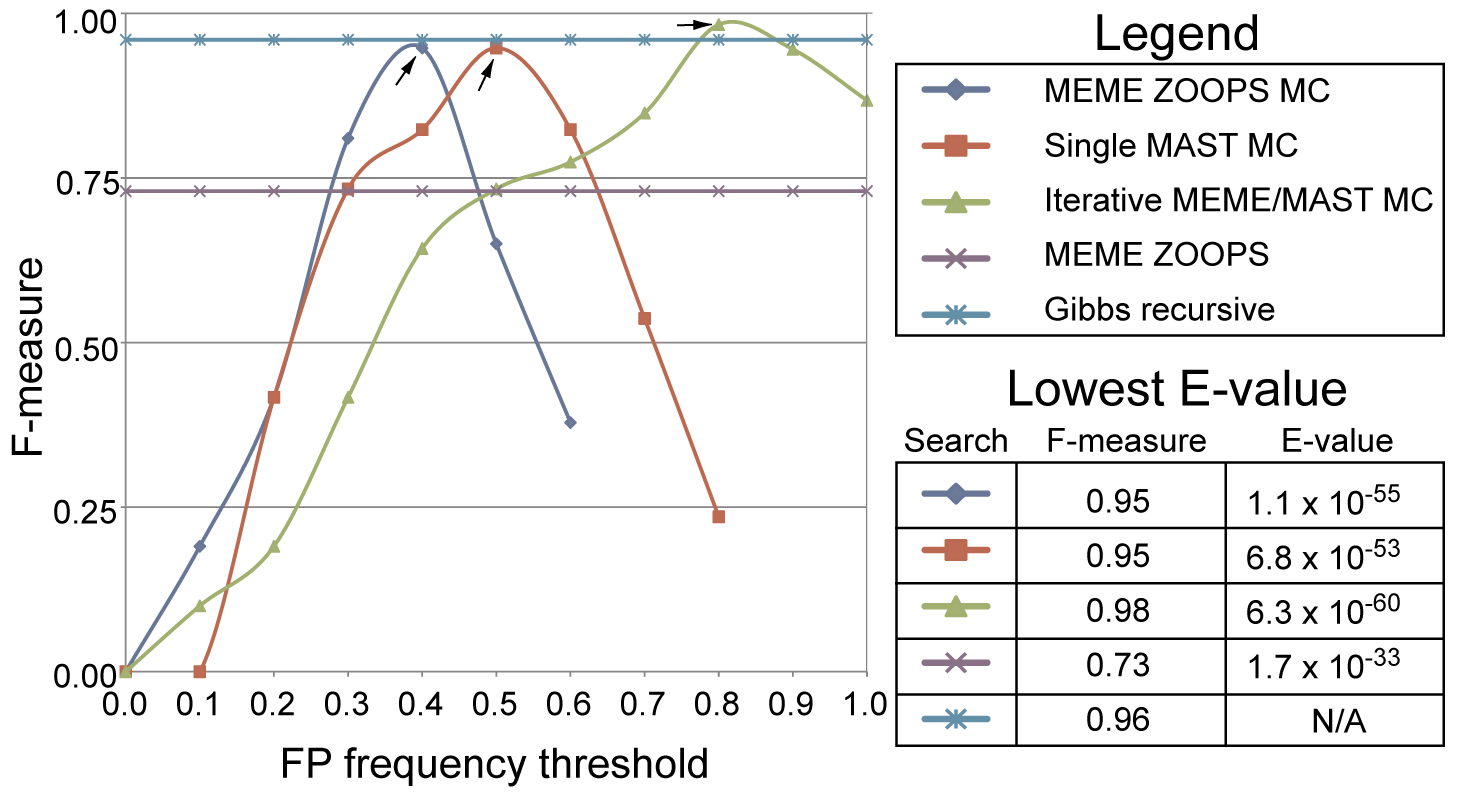


**Supplementary Figure 3: Motif Finder performance on LexA data set as a function of FP frequency threshold**

Plot (left) shows the F-measure (harmonic mean of sensitivity and specificity) for the data set with random sites substituted for type III (unconventional) sites. Results from each of the 3 MotifCatcher search designs (MEME ZOOPS MC, Single MAST MC, and Iterative MEME/MAST MC) are shown at various FP frequency thresholds, and compared to both a MEME ZOOPS and Gibbs recursive sampler search results. The lowest E-value output motif for each MotifCatcher search protocol over the range of all FP frequency threshold values (data points on plot indicated with arrows) always corresponded to the best F-measure for each data set. With this additional specification, all of the MotifCatcher runs (MEME ZOOPS MC, Single MAST MC, and Iterative MEME/MAST MC) significantly outperformed MEME, and demonstrated comparable performance to the recursive Gibbs sampler. Note that the Iterative MEME/MAST MC approach produced candidate motifs over the whole range of FP frequency threshold values, while the MC approaches failed to suggest motifs at thresholds above 0.6 (in the case of the MEME ZOOPS MC search) and 0.8 (in the case of the Single MAST MC search). In general, a rightward shift occurred in the point of the FP frequency threshold maximum; however, this is also a function of the total number of runs performed. The relative shapes of the curves, however, revealed that the Iterative MEME/MAST MC approach had the largest proportion of FP frequency threshold values above the F-measure of the MEME ZOOPS approach, followed by the Single MAST MC search and MEME ZOOPS MC approach. The combination of this observation, coupled with the fact that this search achieved the highest F-measure score of the 3 MC searches, suggests that the Iterative MEME/MAST MC approach demonstrated superior performance to the other two available MC approaches, when applied to the LexA dataset.

S5: Supplementary Excel Spread Sheet

Please see the associated file Supplemental_Data.xls


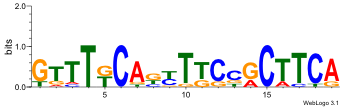


**Supplementary Figure 4: Novel motif discovered in Type III LexA binding sites**

An additional MotifCatcher run was carried out using only the Type III LexA binding sites as the whole input data set. 500 random seeds were taken of size 6, and an iterative MEME/MAST MC search was carried out with a MAST threshold of 0.50. Motifs could be discovered on the forward or reverse strand, and could have a length from 10 to 30 nucleotides. The motif logo shown above is 19 nt long, and was discovered in 8 of the 19 sites. The motif has an E-value of 5.8e-007, relative to the 8 sites from which it is derived. No significant correlation could be determined between the location of the above motif and weak matches to the putative canonical LexA binding site (data not shown).

References

S1. Arndt, K., & Fink, G. R. (1986). GCN4 protein, a positive transcription factor in yeast, binds general control promoters at all 5’ TGACTC 3' sequences. *Proceedings of the National Academy of Sciences of the United States of America*, **83**:22, 8516-20.

S2. Harbison, C. T., Gordon, D. B., Lee, T. I., Rinaldi, N. J., MacIsaac, K. D., Danford, T. W., Hannett, N. M., et al. (2004). Transcriptional regulatory code of a eukaryotic genome. *Nature* 2004, **431**:99.

S3. Gordân, R., Hartemink, A. J., & Bulyk, M. L. (2009). Distinguishing direct versus indirect transcription factor-DNA interactions. *Genome research*, **19**:11, 2090-100. doi:10.1101/gr.094144.109
